# Rural parents’ adherence to infant feeding guidelines to prevent allergy: a cross sectional study in New South Wales

**DOI:** 10.1186/s12889-023-17396-8

**Published:** 2023-12-08

**Authors:** Gianni Rossi, Jessica Cesca, Charmie Fong, Andrew Wallace, Peter Simmons DComm, Uchechukwu Levi Osuagwu, Jannine Bailey, Tegan Dutton, Adambarage Chandima De Alwis

**Affiliations:** 1 Bathurst Rural Clinical School, School of Medicine, Western Sydney University, Sydney, Australia; 2 School of Rural Medicine, Charles Sturt University, Bathurst, Australia; 3 Gulbali Research Institute, Charles Sturt University, Bathurst, Australia; 4 Translational Health Research Institute (THRI), Western Sydney University, Sydney, Australia; 5 School of Medicine, Western Sydney University, Campbelltown, NSW Australia; 6 Department of Paediatrics, Bathurst Base Hospital, Bathurst, NSW 2795 Australia

**Keywords:** Rural, Infants, Allergen, ASCIA guidelines, Awareness, Compliance, Breastfeeding, Peanut, Solids

## Abstract

**Background:**

Responding to international research showing that early introduction of common food allergens can reduce the chance of developing allergies, in 2016 the Australasian Society of Clinical Immunology and Allergy (ASCIA) revised allergen introduction guidelines, recommending earlier introduction of allergens to infants in their first year. Australia has high food allergy rates, and limited understanding of adherence to allergen introduction guidelines, especially in rural areas. This project explored rural parent adherence to ASCIA guidelines.

**Methods:**

This was a mixed method cross sectional study using an online survey including multiple-choice and qualitative short answer responses. The sample were 336 women from two rural health districts in New South Wales. All were aged 18 or over, and either pregnant or had delivered a baby since July 2018. Descriptive statistics were used to measure behavioural alignment with the recommended guidelines, thematic analysis was used to analyse attitudes and explanations.

**Results:**

In 84.3% of children, feeding adhered to all four guidelines studied, including *no elimination of allergens during pregnancy (98%), age of introduction of solids (97.7%), continuation of breast milk/cow’s milk formula during introduction of solids (95%)*, and *age of introduction of allergens (92.9%).* Adherence was not significantly correlated with the education (X^2^ = 17.9, P = .056), prior history of allergy [neither mother (X^2^ = 0.945,P = .623) nor previous children (X^2^ = 0.401,P = .818)], or primary care received during pregnancy. More than 90% of participants agreed or strongly agreed that the guidelines are realistic, trustworthy, and important for the health of their child. However, thematic analysis revealed that parents’ perceptions of a child’s individual progress, and medical conditions or other circumstances, such as challenges with breastfeeding, will often take precedence over adherence to specific guideline recommendations.

**Conclusions:**

High rates of adherence with ASCIA guidelines found here are comparable with findings from metropolitan studies and encouraging for future population health. Participant comments on the guidelines imply to rural policymakers that there are multiple influences on parent decisions about infant feeding, often including parents’ own intuition and experiences. Further studies to improve understanding of the role of information, carers, and other influences on parent decision-making concerning feeding attitudes and behaviours will be necessary to optimise adherence in rural areas.

**Supplementary Information:**

The online version contains supplementary material available at 10.1186/s12889-023-17396-8.

## Background

Australia has one of the highest food allergy rates in the world [1], with more than 10% of one year old infants [2, 3] and 3.8% of 4 year olds [3] having at least one known allergy. The most common food allergens observed in Australia and New Zealand are eggs, cow milk, peanuts, tree nuts, sesame, soy, fish, shellfish, and wheat [4]. An increase in food allergies in many developed countries [1] has led to extensive research into preventing food allergy development. More recent evidence indicates that early introduction of common food allergens into an infant’s diet can reduce the chance of developing allergies later in life [5–7], challenging previous infant feeding guidelines, which advised avoidance of food allergens variously from 10 months to 3 years, depending on the allergen.

With the aims of providing clear guidance to the public while promoting allergy prevention and nutrition priorities [8], the Australasian Society of Clinical Immunology and Allergy (ASCIA) published allergen introduction guidelines in 2016 [9] and then a further update in July 2018 titled “Infant Feeding and Allergy Prevention Clinical Update” [10]. The guidelines outlined the diet recommended for infants in the first year of life to reduce the likelihood of later developing allergies, and there was considerable effort to promote the guidelines in medical and general media [11]. Delays in introducing allergens may be associated with a rise in food allergies [1, 6, 7]. Exploring parent adherence and attitudes towards allergy prevention guidelines may improve understanding of behaviours, which in turn may lead to reducing food allergy rates.

The revised guidelines recommended introduction of eggs and peanut protein prior to 12 months [10]. Limited Australian research since the ASCIA guidelines were introduced suggests relatively high adherence. Melbourne-based studies have shown that in 2017–2019, after the introduction of the ASCIA guidelines and compared with a similar sample from 2007 to 2011, the introduction of peanuts to infants in their first year of life was three times higher at 88% [12], the proportion of infants breastfed for 12 months had increased almost 17–52.7%, and the proportion of infants introduced to solid foods at 4 months had increased more than 12–31.9% [8]. Another study found that 86.2% of infants had eaten foods containing peanut in their first 12 months [11].

However, most Australian studies have been metro-centric and there is very limited published data on rural families and their application of the guidelines for infant feeding, before or since their introduction. This is an important gap in our knowledge for a range of reasons [13]. Adherence can be influenced by personal and community contexts and rural communities often have worse outcomes and lower levels of health literacy than urban communities in the same state or country [14]. One study of mothers’ practices relating to breastfeeding guidelines reported substantial influence on behaviour of different sources of advice, and personal judgements about child needs and the right thing for a mother to do [15]. We addressed this gap in understanding with a study of parents’ attitudes and behaviours relating to infant feeding practices in a rural context in Australia.

### Aims

The study aimed to investigate parents’ adherence to the ASCIA guidelines and barriers to adherence in a large rural area. It asked the following research questions:

- Are women’s practices concerning introduction of foods to their infant adherent with the ASCIA 2018 guidelines?
- What are women’s attitudes toward the guidelines?

It focused on families in the western regions of NSW, Australia.

## Methods

### Study design

This was a mixed methods cross-sectional study conducted using an online survey designed using Qualtrics software XM (North Sydney, NSW, Australia) [16]. Related literature was used to aid the development of the instrument [13, 15, 17–19] which included multiple-choice and qualitative short answer responses (Additional file 1). Most questions asked about feeding practices with infants. Towards the end of the survey a link to the updated 2018 ASCIA guidelines was provided, with questions on attitudes towards the guidelines. General demographic information about the participants (gender, age, postcode, number of children) and family history of allergy were also collected. Data from a pilot test with 10 women used to refine the final instrument were not included in the final analysis.

### Participant eligibility and recruitment

Eligible participants were women aged 18 or over who were pregnant or had a baby since July 2018 (when the ASCIA guidelines were updated) and living within Western or Far Western NSW Local Health District (LHD) regions. These two health districts (Far West LHD; Western LHD) covering more than 441,000 square kilometres, and servicing a total 2021 population of approximately 310, 000 were chosen because they include many rural, remote and very remote communities. Participants had to complete at least 75% of the survey to be included in the analysis. This level of completion was deemed indicative of intention to contribute to the study.

Data was collected between July and October 2021. Due to COVID-19, a predominantly contactless approach was used. Researchers used Facebook to share the survey link with maternal and baby groups and community noticeboard pages in Western NSW and Far West LHD towns listed with local health facilities/hospitals. The link was shared via at least 64 regional and local community social media groups, and survey QR codes were displayed on flyers in health settings.

### Ethics

The survey was anonymous, participants gave informed consent to participate but were able to withdraw from the study until submission of their responses. Ethics approval was obtained from the Western Sydney University Human Research Ethics Committee (Approval H14301). The Australian Breastfeeding Association also approved the survey and posted links to the survey on their relevant Facebook pages (Approval 2021-13).

### Data analysis

Statistical analysis was performed using the Jamovi Version 2.2.2 [20]. Descriptive statistics were used to describe the survey participants. Yes/no and multiple-choice questions were used to determine alignment of women’s current feeding practices with recommended guidelines as shown in Table 1.

**Table 1:** Classification of guideline adherence levels

Guideline	Adherent	Non-adherent
Age of introduction of solids	4–6 months, 7–9 months	0–3 months, 10–12 months, 12 + months
Continuation of breast milk/cow’s milk formula during introduction of solids	Milk continued	Milk not continued
Age of introduction of allergens	0–6 months, 7–12 months	12 + months
Consume common allergens during pregnancy*	Not restricted	Restricted

*Not restricted is defined as women who consumed 3 or more common allergens in pregnancy. Restricted is defined as women who consumed 2 or fewer common allergens in pregnancy

For the quantitative data, each respondent was given a simple ‘adherence’ percentage score as follows. Adherent to all 4 items (100%), 3 items (75%), 2 items (50%), 1 item (25%), and 0 item (0%). ‘Adherence’ is the term applied throughout the study to people whose behaviours were consistent with the guidelines. However, some of those recorded as ‘adhering’ or not adhering will have done so for reasons not related to allergy prevention or the guidelines and recommendations. (For example, vegans and vegetarians restrict diet for reasons other than allergy.) Quantitative analysis involved identifying alignment of feeding practice variables with the 2018 ASCIA guidelines and correlations with the demographic variables provided. Chi square test was used to investigate associations between the adherence score differences and the demographic variables.

Qualitative data were manually analysed to identify themes in thinking or meaning in the content of responses [21] to questions about the credibility and usefulness of the guidelines. Saliency of themes reported in part B was partially informed by the frequency of theme mentions.

## Results

A total of 468 surveys were completed, after removing 10 underage, those outside the target health districts (n = 37), and less than 75% completion (n = 85), 336 were deemed eligible, see Table 2. The age breakdown of the all-female respondents was similar to NSW mothers: 18–20 (1.5% vs. NSW under 20, 1.4%), 21–25 (7.1% vs. NSW 20–24, 9.1%), 26–30 (31.3% vs. NSW 25–29 24.6%), 31–35 (37.8% vs. NSW 30–34, 37.5%), 36 plus (22.3% vs. NSW 35 plus 27.4%) [22]. At the time of the study 72% (240) had one child born since July 2018, 19% (67) had two children or more, and 8% (26) were pregnant and without children when completing the survey. 33% of respondents reported that either they or their partners have an allergy.

**Table 2:** Sample characteristics

Age years (n,%)	Occupation (n,%)	Occupation (n,%)
n = 336	n = 316	
18–20 (5,1.48)	Public Admin/Safety 8 (2.53)	Mining 6 (1.89)
21–25 (24,7.14)	Administration 32 (10.12)	Scientific/tech 14 (4.43)
26–30 (105,31.25)	Agriculture/forest/ Fish 17 (5.37)	Arts/Recreation 5 (1.58)
31–35 (127,37.79)	Information/Communications 6 (1.89)	Retail 17 (5.37)
36+ (75,22.32)	Education/Training 67 (21.2)	Hospitality 11 (3.48)
	Finance/Insurance 12 (3.79)	Unemployed 31 (9.81)
	Healthcare/Social 83 (26.26)	Other (2.19)
**Highest education** (n,%)	**Parents with allergies** (n,%)	**Children with allergies** (n,%)
n = 323	n = 323	n = 323
Year 10 (12,3.71)	Yes (109,33.74)	Yes (64,19.81)
Year 12 (43,13.31)	No (214, 66.25)	No (259,80.18)
TAFE (89,27.72)		
Bachelor or higher (188 (58.2)		

The sample were drawn from 50 different NSW postcodes. The majority of the respondents were Australian born and English was the primary language spoken at home by all but one respondent (Croatian) (vs. NSW 68% English at home) [23]. More than half (58.2% vs. NSW, 27.8%) had a bachelor’s degree or higher [23], and 26.2% worked in the health sector, followed by those in education (21.2%).

### Adherence to ASCIA guidelines

Table 3 shows high levels of adherence to the guidelines. All participants adhered to at least two of the four recommendations from the guideline, with 97% adhering to at least three of the recommendations.

**Table 3:** Participant adherence to ASCIA guidelines 2018

	Adherence			
**Adherence level**	Currently pregnant n = 60 (%)	First eligible child n = 323 (%)	Second eligible child n = 64 (%)	All Children N = 447 (%)
100% (all 4 guidelines)	50 (83.3)	278 (86.1)	49 (76.5)	377 (84.3)
75% (3 guidelines)	10 (16.7)	38 (11.8)	12 (18.8)	60 (13.4)
50% (2 guidelines)	0	7 (2.2)	3 (4.7)	10 (2.2)

Table 4 presents the proportion of subgroups of women with different numbers of children who were adherent and non-adherent to each of the guidelines. Table 4 uses the total of 447 (respondent reports of children already born and in utero) as the denominator. The highest total adherence was to advice to consume common allergens during pregnancy (98%). This was followed by advice to introduce solids between 4 and 9 months (97.7%). Lowest total adherence was to advice to introduce allergens before 12 months (92.9%). For pregnant women and women reporting their first child, the lowest adherence was to the recommended age of introduction of allergens. For women with two eligible children, the lowest adherence (10.9%) concerned continuation of breast/cow’s milk during introduction of solids.

**Table 4:** Adherence and non-adherence to different guidelines by sub-group

	Non-adherence			Adherence
**Recommendation in guideline**	Currently pregnant n = 60 (%)	First eligible child n = 323 (%)	Second eligible child n = 64 (%)	All children Adherence n = 447(%)
Consume common allergens during pregnancy	2 (3.3)	6 (1.9)	1 (1.6)	438 (98)
Age of introduction of solids	2 (3.3)	6 (1.9)	2 (3.1)	437 (97.7)
Continuation of breast milk/cow’s milk formula during introduction of solids	2 (3.3)	11 (3.4)	7 (10.9)	427 (95)
Age of introduction of allergens	4 (6.6)	22 (6.8)	5 (7.8)	416 (92.9)

Participant education level (X^2^ = 17.9,P = .056) had a low association with levels of adherence to the ASCIA guidelines, but the study found no evidence of association between participants’ occupation, primary care received during pregnancy, including obstetrician (X^2^ = 3.52,P = .172), nurse (X^2^ = 0.263,P = .877), midwife (X^2^ = 2.31,P = .315), or GP (X^2^ = 4.7,P = .095), and different levels of adherence to the guidelines. Women already with and without children with allergies (X^2^ = 0.401,P = .818), and women with and without allergies themselves (X^2^ = 0.945,P = .623), were similarly adherent to the guidelines.

Participants were asked if feeding practices had changed for children born prior to July 2018 and children born after July 2018. One hundred and thirty (38.7%) checked ‘I have no children born previous to July 2018’. Twenty-nine (8.6%) replied that they had changed, and were asked to comment on why. Their responses are summarised in Table 5.

**Table 5:** Main reasons for the differences in feeding practices between children born to the same mother

Theme	Frequency	Example quote
Current maternal or child medical condition **Subthemes**: Baby swallowing difficulties (2) Morning sickness (2) Allergies in child (2) Maternal allergy (1) Premature birth (1) Developmental concerns (1) Unspecified (1)	10	I started to react to nuts and milk during pregnancy number two.
Increased knowledge / confidence with later children	5	1st child was 10 years earlier and I was a lot younger
Breastfeeding difficulty	4	I solid fed my 1st child at 4 months due to failure to thrive on breastmilk
Allergy in previous child	3	Oldest was allergic to dairy… I have avoided exposing my youngest to dairy via my breastmilk this time and so far he has zero allergies or eczema.
Received external advice (e.g. midwife)	2	First child - solids at 4–5 months at recommendation of midwife in attempt to settle fussy baby as they felt he was hungry…
Followed child’s cues which were different to previous child	2	My 2nd born stopped feeding much earlier at 9 months compared to me stopping my 1st born at 20 months
Social / personal barriers	2	Didn’t breast feed for long with first two kids as I had no support to help me. Closest help was 2 h away

There were 28 open-ended responses to the question about why feeding practices had changed. The most common responses indicated medical conditions affecting either the mother or child (35%), followed by increased knowledge/confidence with subsequent/future children (17.9%), and difficulty with breastfeeding (14.3%).

### Attitudes towards ASCIA guidelines

Participants were given a summary of, and link to, the guidelines, and asked to rate their level of agreement with several statements (Table 6 column 1) about achievability and importance, and trust in the guidelines. Attitudes towards the guidelines tended to be very positive, 91% of participants agreed or strongly agreed they would tell others about ASCIA, and 97% of participants believed that the guidelines were realistic and achievable. Table 6 (second column from left) shows that almost all agreed or strongly agreed the guidelines were important for the health of their child (96%), that they would trust information from ASCIA (97%), and that they would follow the ASCIA guidelines after hearing about them (96%).

Participants were invited to comment on each statement concerning the 2018 ASCIA infant feeding guidelines. Table 6 column 3 shows the main themes people commented on, column 4 shows how many comments on that statement related to the theme, and column 5 gives an example of a comment that illustrates the theme.

**Table 6:** Responses to the guidelines

1 Statement (and number of respondent comments)	2 Agree/Strongly agree %	3 Main themes	4 Frequency of comment theme / total comments	5 Essential quote
These guidelines are realistic and achievable (33 comments)	97	Depends on the individual child	12 / 33	I believe that it’s important to communicate to families that 4–6 months is a guide. They should be encouraged to look at the cues a child provides as to when they are ready for solids.
		Depends on ability to breastfeed	9 /33	I know breastfeeding isn’t possible for all family situations and dynamics nor can all females breastfeed successfully
Following the guidelines is important for the health of my child (14 comments)	96	Following the child’s progress is most important	10 / 14	Every child is different. What could or does work for one may not work for others
		Unhappy with guideline focus on breastfeeding over formula	3 /14	I was unable to breastfeed due to unknown issues… so the guidelines consistently make me feel so upset because I tried to do the right thing for my child according to the guidelines, but it didn’t eventuate.
I would follow these guidelines after hearing about them (7 comments)	96	Depends on child’s individual progress	5 /7	I believe each child is individual. Guidelines are great but cannot always be followed closely.
		Perceived critical tone of writing in guidelines may deter some people (especially with regards to breastfeeding)	2 /7	Women are told regularly what to do in pregnancy and there is so much conflicting information available. I think for these guidelines to be effective; the design of information must be expandable. For example, therefore we recommend this. if you have any questions or concerns speak with your GP. So many women seek other sources of information that align with their own ideas or ethos. It is important that the tone of voice within the guidelines is sensitive to this and allows those mothers to feel not criticized for those actions but rather encouraged to make a change for the betterment of their children.
I would trust information from the Australasian Society of Clinical Immunology and Allergy. (10 comments)	97	Needs additional information regarding ASCIA	5 /10	I feel inclined to trust them, but at this stage I still don’t know anything about them. I know this guideline is the same as other sources I trust. I can’t say if I would equally trust any of their other guidelines - I don’t know their process for research and recommendations.
		Needs more focused education and promotion of guidelines	2 /10	Mothers, and particularly first-time mothers, are bombarded with information so it is often hard to distinguish between what is important and imperative information and what is so-so.
		Would use ASCIA information only in conjunction with their healthcare provider	2 /10	I feel that they may not be as up to date and definitely not specific to my child’s needs. Therefore I use a pediatrician.

Although there was general confidence in ASCIA and the guidelines, not all considered the recommendations suitable for their situation. The most common concern reported by the women about the achievability of the guidelines was that this depended on the needs of the individual child. A further nine women said that it was dependent on the ability to breastfeed.

The majority of the 14 comments on the importance of the guidelines for the health of their child emphasised the progress of each individual child. Three women were also unhappy with the specific focus of the guidelines on breastfeeding rather than formula feeding. Among those who responded to the statement “*I would follow these guidelines after hearing about them”*, five emphasised their child’s individual progress taking precedence over the specific guideline recommendations. A further two responses stressed the importance of communicating in an appropriate tone, especially with respect to breastfeeding, which is not achievable by all.

Of the 10 comments obtained on the statement *“I would trust information from the Australasian Society of Clinical Immunology and Allergy”*, half were unsure or needed further information. Others commented on the need to communicate clearly through the large amount of information targeting new mothers, and their preference for working through a healthcare provider.

Participants were also asked if they were aware of other infant guidelines, and to list them. Sixty-five (19.3%) said they were aware of other guidelines, among them, 13 respondents specified guidelines. Eight referred to World Health Organisation (WHO) guidelines, and two referred to Australian Breastfeeding Association guidelines.

## Discussion

The findings for this rural sample show a large majority adhered to the recommended feeding practices intended to reduce the likelihood of developing food allergies [11], and the results were similar to other Australian studies. The finding that more than 9 in every ten women in the study said they had introduced or would introduce allergens in the first year of life is similar to recent Australian metropolitan studies [11, 12] of the introduction of peanuts in the first 12 months. Further, the finding that nearly all the women in this study reported introducing solids when children were between four and nine months accords with another Australian metropolitan (EarlyNuts) study from 2017 to 2019 that reported 92.6% introducing solids between 4 and 7 months [8]. This rural study found that a higher proportion of women (95%) continued breastfeeding or using cow’s milk formula after the introduction of solids than the 81.9% of the EarlyNuts sample who were breastfed for more than 4 months, and 52.7% for more than 12 months [8].

This study reports a sample that is more highly educated than the NSW average. We found no significant association between sociodemographic factors (e.g. education, current occupation) or source of primary care received during pregnancy, and rates of adherence with the current infant feeding guidelines. While our study found no association between parental allergy and adherence to the ASCIA guidelines, this finding conflicts with previous studies that have found associations. A 2021 US study [24] found that women with existing food allergies were more likely to introduce key food allergens by 12 months than women without food allergy. In a Victorian cohort study conducted in 2014 [25] an absence of family allergies led to better adherence to the then updated 2008 ASCIA guidelines. In that study, mothers who were born outside of Australia and those from higher socioeconomic status were more likely to adhere to the guideline [25]. A future study should be designed to improve understanding of ways that family experience of anaphylaxis and knowledge of allergy influence caution and behaviours relating to infant feeding practices.

Participants’ written comments indicated generally very positive attitudes towards the ASCIA guidelines. Respondents felt that following the guidelines was important for the health of their child and that they would trust and share information from ASCIA. However, they also emphasised the importance of tailoring feeding practices to each child and situation, such that the ease and importance of following the guidelines is dependent on the child’s progress and needs. Practices that differed from the guideline varied, most were justified by the mother’s or child’s medical needs, including morning sickness, allergies, swallowing difficulties, or failure to thrive.

Another frequent and important theme was that not all women are in a position to adhere to the guidelines, especially considering the challenges some experienced with breastfeeding. Similar to the findings of a 2019 qualitative analysis on Australian mothers’ understanding of infant feeding guidelines [15], other themes of note included access to numerous sources of information, which could be conflicting, and the notion that the guidelines change too frequently, leading to confusion.

Comments emphasised the importance of sensitive communication, appropriate in content and expectation of women, and avoiding tones that might make women feel inadequate or guilty. Some feel strongly that women who are unable to breastfeed should not be diminished or made to feel inadequate.

Information about recommended infant feeding practices reaches women from numerous sources including professionals such as maternal and child health nurses, general practitioners and paediatricians, informally from family and friends, and also through various media, with and without explicit connection to ASCIA or guidelines.

Agreement on preferred wording and communication of information related to the timing of introducing solid foods and inclusion of allergens in the diet [26] may maintain and improve high adherence levels. The effectiveness of campaigns targeting the allergy epidemic such as “Nip allergy in the bub”, specifically in rural areas, and its role in improving adherence to the ASCIA guidelines should also be further explored.

In the absence of directly comparable studies, we recommend a future study including urban and rural samples for better insight into communication of, attitudes towards, and adherence to the ASCIA guidelines. This would provide valuable insights for targeted communication of the guidelines.

### Strengths and limitations

Several features of the study design might be expected to produce a sample more focused on infant care practices, and compliant with guidelines, than a truly representative sample of rural mothers. The sample was a convenience sample and may not be statistically representative of the target population of mothers [13]. 33% of respondents reported that either they or their partner have an allergy, this figure would be high for individuals, but not necessarily for two people. The contactless distribution of the survey through social media enabled engagement with a wider range of communities than would have been possible in person, including access through parent groups, community notice boards, leisure centres, schools, newspapers and spiritual groups. However, this online approach also comes with some disadvantages that should be considered when interpreting the findings. It prevented reach to communities and subgroups not using social media or connected to health facilities or parent groups. The sample here was more highly educated than a randomly selected sample. In this sample 58% reported a bachelor’s degree or higher (NSW, 27.8%) [22]. However some of this difference may be explained by use of fewer qualification categories in this study than collected in a census, people in our study had no option to specify diplomas and advanced diploma qualifications [27]. Unfortunately the online distribution method used here prevented understanding of which eligible participants exactly were reached but chose not to complete and respond to the survey.

The sample who agreed to the request to complete the survey may have been inherently biased towards people generally inclined to comply. The retrospective self-report collection of data may have led to recall or social desirability bias, and the use of questions assessing intended feeding practices may not be an accurate reflection of behaviours. Women had more and less recent experience of infant parenting due to the different ages of their children, this may have affected the accuracy of their recall of the guidelines and their own behaviours and attitudes. Further, the survey was distributed through the Australian Breastfeeding Association which may have contributed to high engagement and higher than average compliance.

The study reports findings both from people reporting experiences of infant feeding, and people who were pregnant and reporting intentions. The study finds and concludes that infant feeding is subject to multiple influences, and the infant feeding intentions reported may differ from actual practices. The questions focused on source of pregnancy care, but postnatal care may be even more pertinent influences on infant feeding. Future studies should collect information about sources of postnatal care, and other influences on infant feeding decisions. This study used Chi square analysis to examine relationships among variables. Future studies should consider use of logistic regression analysis which would enable adjustment for variables that may confound the relationships being examined.

This study used unique questions, and an innovative scoring scale intended to gauge levels of adherence, that were not always directly comparable with other studies. Future studies should use or develop standardised survey instruments, target inclusion of socioeconomically or otherwise disadvantaged subgroups, and include remote, regional and metropolitan women.

## Conclusion

This study contributes new insights into infant feeding attitudes and practices among recent or expecting mothers in rural NSW. The finding of high rates of compliance similar to metropolitan studies is encouraging for future protection against several common allergens, while mothers’ comments about the guidelines and practices are a reminder that there are multiple influences on critical decisions about infant feeding. Comments on the guidelines remind us that decisions are complex, resulting from active and passive information-seeking, intuition and experiences concerning their own children, and advice from, and trust in, different professional carers. Some remain non-adherent to the guidelines, and future studies exploring the role of information and other influences on parent decision-making concerning feeding attitudes and behaviours, in rural and metropolitan areas, will be necessary to optimise adherence.

### Electronic supplementary material

Below is the link to the electronic supplementary material.

Supplementary Material 1

## Data Availability

The datasets generated and/or analysed during the current study are available in the **Research Direct** repository, https://research-data.westernsydney.edu.au/published/ecffc430ee2c11edb7526f981868d835.

## Abbreviations

ASCIA: Australasian Society of Clinical Immunology and Allergy
NSW: New South Wales (one of seven states of Australia)
WHO: World Health Organisation
