# Supplementary material for: Rural parents’ adherence to infant feeding guidelines to prevent allergy: a cross sectional study in New South Wales
Source: BMC Public Health. 2023 Dec 8;23:2458. doi: 10.1186/s12889-023-17396-8 (PMC10704778; doi:10.1186/s12889-023-17396-8)
Supplement: Supplementary file 1 — Supplementary Material 1 [file 12889_2023_17396_MOESM1_ESM.docx]

**Additional file 1: Questionnaire**

**About you**

This section is about you

Q7: Current age

- 18-20
- 21-25
- 26-30
- 31-35
- 36+

Q8: Gender

- Male
- Female
- Non-binary/third gender
- Prefer not to say

Q9: Country of Birth - __________________________

Q10: Current home postcode - _________________________

Q11: How long have you lived here?

- 1 year or less
- 2-5 years
- 6-10 years
- 11-15 years
- 16-20 years
- 20+ years

Q12: Primary language spoken at home – ____________________

Q13: Occupation

- Accommodation and food services
- Administrative and support services
- Arts and recreation services
- Agriculture, forestry and fishing
- Construction
- Education and Training
- Electric, gas, water and waste services
- Financial and insurance services
- Health care and social assistance
- Information media and telecommunications
- Manufacturing
- Mining
- Professional, scientific and technical services
- Public administration and safety
- Rental, hiring and real estate services
- Retail trade
- Transport, postal and warehousing
- Wholesale trade
- Other

Q14: Highest level of education attained

- Year 10
- Year 12
- TAFE
- Bachelor’s
- Master’s
- PhD

**General information on children and pregnancies**

Q15: How many children do you have?

- 0
- 1
- 2
- 3
- 4
- 5
- 6 or more

Q16: How many children do you have that were born on or after July 2018?

- 0
- 1
- 2
- 3
- 4 or more

Q17: Are you or your partner currently pregnant?

- Yes
- No

Q18: Do you have a regular General Practitioner (GP)?

- Yes
- No

Q19: Do you or your partner have eczema?

- Yes
- No

Q20: Do you or your partner have allergies?

- Yes
- No

Q21: Please specify what allergies – ________________

Q22: Do any of your children have eczema?

- Yes
- No

Q23: Do any of your children have allergies?

- Yes
- No

Q24: Please specify what allergies – __________________

**Current Pregnancy**

The following section is in reference to your current pregnancy only.

Q25: Who is the main source or sources of medical care during you or your partner's pregnancy? Please select all that apply.

- Obstetrician and Gynaecologist (Ob/Gyn)
- General Practitioner (GP)
- Midwife
- Nurse
- Other

Q26: Which of the following do you intend to feed your child:

- Breast milk
- Formula
- Both

Q27: Up to what age will your child be fed solely breast milk and/or formula? Please select all that apply.

- 0-3 months of age
- 4-6 months of age
- 7-9 months of age
- 10-12 months of age
- 13+ months of age

Q28: At what age will your child be **first introduced** to solid foods?

- 0-3 months of age
- 4-6 months of age
- 7-9 months of age
- 10-12 months of age
- 13+ months of age

Q29: Do you intend on continuing breast or formula feeding whilst solid foods are introduced?

- Yes
- No

Q30: At what age will your child first be introduced to common food allergens? (e.g. peanuts, tree nuts, cow’s milk, egg, wheat, soy, sesame, fish, shellfish)

- 0-6 months of age
- 7-12 months of age
- 13-18 months of age
- 19-24 months of age
- 25-36 months of age
- 37+ months of age
- I do not plan to introduce my child to common food allergens

Q31: If allergens will be introduced at various ages, please elaborate: _____________________

Q32: Did/will you consume common food allergens during pregnancy? (e.g. peanuts, tree nuts, cow’s milk, egg, wheat, soy, sesame, fish, shellfish)

- Yes
- No

Q33: Please specify what food allergens you have/will consume during your pregnancy: (e.g. peanuts, tree nuts, cow’s milk, egg, wheat, soy, sesame, fish, shellfish) ______________________

**For your first/only child born during/after July 2018:**

This section is in reference to your first child born on or after July 2018 only.

Q34: Who was the main source or sources of medical care during you or your partner's pregnancy? Please select all that apply.

- Obstetrician and Gynaecologist (Ob/Gyn)
- General Practitioner (GP)
- Midwife
- Nurse
- Other

Q35: How old is your child currently?

- 0-3 months of age
- 4-6 months of age
- 7-9 months of age
- 10-12 months of age
- 13-24 months of age
- 25+ months of age

Q36: Was/is your child fed:

- Breast milk
- Formula
- Both

Q37: Up to what age was/will your child be fed solely breast milk (or formula if not possible)? Please select all that apply.

- 0-3 months of age
- 4-6 months of age
- 7-9 months of age
- 10-12 months of age
- 13+ months of age

Q38: At what age was/will your child be **first introduced** to solid foods?

- 0-3 months of age
- 4-6 months of age
- 7-9 months of age
- 10-12 months of age
- 13+ months of age

Q39: Will/did you continue breast or formula feeding whilst solid foods were introduced?

- Yes
- No

Q40: At what age was/will your child **first be introduced** to common food allergens? (e.g. peanuts, tree nuts, cow’s milk, egg, wheat, soy, sesame, fish, shellfish)

- 0-6 months of age
- 7-12 months of age
- 13-18 months of age
- 19-24 months of age
- 25-36 months of age
- 37+ months of age
- I do not plan to introduce my child to common food allergens

Q41: If allergens were/will be introduced at various ages, please elaborate: ___________________

Q42: Did you consume common food allergens during pregnancy? (e.g. peanuts, tree nuts, cow’s milk, egg, wheat, soy, sesame, fish, shellfish)

- Yes
- No

Q43: Please specify what food allergens you consumed during your pregnancy: (e.g. peanuts, tree nuts, cow’s milk, egg, wheat, soy, sesame, fish, shellfish) ___________________________

Q44: Did your feeding practices for children **prior to July 2018** differ to the answers provided above?

- Yes
- No
- I have no children born previous to July 2018

Q45: If yes, please explain why ________________________________

**For your second child born after July 2018**

This section is in reference to your second child born on or after July 2018 only.

Q46: Who was the main source or sources of medical care during you or your partner's pregnancy? Please select all that apply.

- Obstetrician and Gynaecologist (Ob/Gyn)
- General Practitioner (GP)
- Midwife
- Nurse
- Other

Q47: How old is your child currently?

- 0-3 months of age
- 4-6 months of age
- 7-9 months of age
- 10-12 months of age
- 13-24 months of age
- 25+ months of age

Q48: Was/is your child fed:

- Breast milk
- Formula
- Both

Q49: At what age was/will your child be fed solely breast milk (or formula if not possible)?

- 0-3 months of age
- 4-6 months of age
- 7-9 months of age
- 10-12 months of age
- 13+ months of age

Q50: At what age was/will your child be **first introduced** to solid foods?

- 0-3 months of age
- 4-6 months of age
- 7-9 months of age
- 10-12 months of age
- 13+ months of age

Q51: Will/did you maintain breast feeding whilst solid foods were introduced?

- Yes
- No

Q52: At what age was/will your child **first be introduced** to common food allergens? (e.g. peanuts, tree nuts, cow’s milk, egg, wheat, soy, sesame, fish, shellfish)

- 0-6 months of age
- 7-12 months of age
- 13-18 months of age
- 19-24 months of age
- 25-36 months of age
- 37+ months of age
- I do not plan to introduce my child to common food allergens

Q53: If allergens were/will be introduced at various ages, please elaborate: ____________________

Q54: Did you consume common food allergens during pregnancy? (e.g. peanuts, tree nuts, cow’s milk, egg, wheat, soy, sesame, fish, shellfish)

- Yes
- No

Q55: Please specify what food allergens you consumed during your pregnancy: (e.g. peanuts, tree nuts, cow’s milk, egg, wheat, soy, sesame, fish, shellfish) ___________________________

Q56: Did your feeding practices for **prior** children differ to the answers provided above?

- Yes
- No

Q57: If yes, please explain why _____________________________

**For your third child born after 2018**

This section is in reference to your third child born on or after July 2018 only.

Q58: Who was the main source or sources of medical care during you or your partner's pregnancy? Please select all that apply.

- Obstetrician and Gynaecologist (Ob/Gyn)
- General Practitioner (GP)
- Midwife
- Nurse
- Other

Q59: How old is your child currently?

- 0-3 months of age
- 4-6 months of age
- 7-9 months of age
- 10-12 months of age
- 13-24 months of age
- 25+ months of age

Q60: Was/is your child fed:

- Breast milk
- Formula
- Both

Q61: At what age was/will your child be fed solely breast milk (or formula if not possible)? Please select all that apply.

- 0-3 months of age
- 4-6 months of age
- 7-9 months of age
- 10-12 months of age
- 13+ months of age

Q62: At what age was/will your child be **first introduced** to solid foods?

- 0-3 months of age
- 4-6 months of age
- 7-9 months of age
- 10-12 months of age
- 13+ months of age

Q63: Will/did you maintain breast feeding whilst solid foods were introduced?

- Yes
- No

Q64: At what age was/will your child **first be introduced** to common food allergens? (e.g. peanuts, tree nuts, cow’s milk, egg, wheat, soy, sesame, fish, shellfish)

- 0-6 months of age
- 7-12 months of age
- 13-18 months of age
- 19-24 months of age
- 25-36 months of age
- 37+ months of age
- I do not plan to introduce my child to common food allergens

Q65: If allergens were/will be introduced at various ages, please elaborate: __________________

Q66: Did you consume common food allergens during pregnancy? (e.g. peanuts, tree nuts, cow’s milk, egg, wheat, soy, sesame, fish, shellfish)

- Yes
- No

Q67: Please specify what food allergens you consumed during your pregnancy: (e.g. peanuts, tree nuts, cow’s milk, egg, wheat, soy, sesame, fish, shellfish) _____________________

Q68: Did your feeding practices for **prior** children differ to the answers provided above?

- Yes
- No

Q69: If yes, please explain why ___________________

**The 2018 ASCIA (Australian Society of Clinical Immunology and Allergy) Guideline**

The 2018 ASCIA (Australian Society of Clinical Immunology and Allergy) Guidelines for Infant Feeding and Allergy Prevention suggest specific practices regarding breastfeeding/formula and solid foods that may help to reduce the risk of infants developing allergic diseases such as eczema and food allergy.

Q70: Have you heard of this guideline?

- Yes
- Maybe
- No

Q71: Are you aware of what this guideline suggests?

- Yes
- Maybe
- No

Q72: If you did not follow these guidelines, please elaborate why: _______________________

Q73: Where did you find out about this guideline? (Select all that apply)

- Health Professional
- Community Group
- Family
- Friends
- Internet
- News
- Others

Q74: Are you aware of any other guidelines in regards to infant feeding and allergy prevention?

- Yes
- No

Q75: Please list the guidelines you know ____________________________

Q76: Where did you find out about these guidelines? (Select all that apply)

- Health Professional
- Community Group
- Family
- Friends
- Internet
- News
- Others

**Opinions towards ASCIA guidelines**

A summary of the ASCIA Guidelines is provided below*:

Breastfeeding alone is recommended for approximately the first 6 months (or standard cow's milk based formula if breastfeeding is not possible).

It is recommended that breastfeeding be continued along with other foods for as long as the mother and infant wish to continue.

When the infant is ready, at around 6 months (but not before 4 months), a variety of solid foods should be gradually introduced (including the common allergens), preferably while continuing to breastfeed. 

**Please note, for more information please seek the ASCIA Guidelines (linked below) or your local healthcare provider.*

<https://www.allergy.org.au/images/stories/pospapers/ASCIA_HP_Clinical_Update_Infant_Feeding_and_Allergy_Prevention_July2018.pdf>  

The following questions are to assess your personal views and opinions towards the ASCIA Guidelines.

Q77: These guidelines are realistic and achievable

| Strongly Disagree | Somewhat Disagree | Somewhat Agree | Strongly Agree |
| --- | --- | --- | --- |
|  |  |  |  |

Q78: Any comments you would like to make: _________________

Q79: Following the guidelines is important for the health of my child

| Strongly Disagree | Somewhat Disagree | Somewhat Agree | Strongly Agree |
| --- | --- | --- | --- |
|  |  |  |  |

Q80: Any comments you would like to make: _________________

 Q81: I would follow these guidelines after hearing about them

| Strongly Disagree | Somewhat Disagree | Somewhat Agree | Strongly Agree |
| --- | --- | --- | --- |
|  |  |  |  |

Q82: Any comments you would like to make: ________________

I would trust information from the Australasian Society of Clinical Immunology and Allergy.

| Strongly Disagree | Somewhat Disagree | Somewhat Agree | Strongly Agree |
| --- | --- | --- | --- |
|  |  |  |  |

Q83: Any comments you would like to make: ________________

Q84: I would tell others about these guidelines

| Strongly Disagree | Somewhat Disagree | Somewhat Agree | Strongly Agree |
| --- | --- | --- | --- |
|  |  |  |  |
